# Optimising the selection of outcomes for healthy ageing trials: a mixed methods study

**DOI:** 10.1007/s11357-022-00690-5

**Published:** 2022-11-17

**Authors:** Muslim Abbas Syed, Olalekan Lee Aiyegbusi, Eliot Marston, Janet M. Lord, Harriet Teare, Melanie Calvert

**Affiliations:** 1 UK SPINE, University of Birmingham, Birmingham, UK; 2 Centre for Patient Reported Outcomes Research, Institute of Applied Health Research, University of Birmingham, Birmingham, UK; 3 Birmingham Health Partners Centre for Regulatory Science and Innovation, University of Birmingham, Birmingham, UK; 4 NIHR SRMRC, University Hospitals Birmingham NHS Foundation Trust and University of Birmingham, Birmingham, UK; 5 NIHR Birmingham Biomedical Research Centre, University Hospitals Birmingham NHS Foundation Trust and University of Birmingham, Birmingham, UK; 6 NIHR Applied Research Collaboration West Midlands, University of Birmingham, Birmingham, UK; 7 NIHR Birmingham-Oxford Blood and Transplant Research Unit (BTRU) in Precision Transplant and Cellular Therapeutics, University of Birmingham, Birmingham, UK; 8 MRC-Versus Arthritis Centre for Musculoskeletal Ageing Research, Institute of Inflammation and Ageing, University of Birmingham, Birmingham, UK; 9 UK SPINE, University of Oxford, Oxford, UK

**Keywords:** Healthy ageing, Population ageing, Outcome measures, Healthy ageing trials

## Abstract

Advancing age is associated with chronic diseases which are the largest cause of death and disability in developed countries. With increasing life expectancy and an ageing population, there is a need to conduct trials to extend healthy ageing, including targeting biological ageing processes, and prevent ageing-related diseases. The main objectives of the study are as follows: (i) to review outcome measures utilised in healthy ageing trials focusing on pharmacological therapies, nutritional supplements and medical devices; (ii) to summarise the views of key stakeholders on outcome selection for healthy ageing trials. An analysis of records from the Clinicaltrials.gov database pertaining to healthy ageing trials from inception to May 2022 was conducted. In addition, the findings of a workshop attended by key stakeholders at the 2022 annual UKSPINE conference were qualitatively analysed. Substantial heterogeneity was found in the interventions evaluated and the outcomes utilised by the included studies. Recruitment of participants with diverse backgrounds and the confounding effects of multi-morbidity in older adults were identified as the main challenges of measuring outcomes in healthy ageing trials by the workshop participants. The development of a core outcome set for healthy ageing trials can aid comparability across interventions and within different settings. The workshop provided an important platform to garner a range of perspectives on the challenges with measuring outcomes in this setting. It is critical to initiate such discussions to progress this field and provide practical answers to how healthy ageing trials are designed and structured in the future.

## Introduction

Population ageing is an important public health challenge [1]. Globally, it is estimated by year 2050 there will be 392 million people aged 80 years or above [2]. There are serious health implications associated with this projected demographic change in the next few decades [3]. Ageing is associated with chronic diseases which are the largest cause of death and disability in developed countries [4, 5] as well as resulting in reduced quality of life of individuals [6]. For instance, ageing is considered as a primary risk factor for neurodegenerative diseases such as Alzheimer’s and Parkinson’s disease which are associated with disability and reduced functionality in daily activities [7]. Moreover, there are serious issues of health inequalities amongst older adults which are mainly attributed to ethnicity, gender and socio-economic position [8–10].

Current research pertaining to ageing mainly entails exploring the biological mechanisms of ageing to better understand the factors associated with age-related diseases to improve health amongst older age groups [11, 12]. The increased knowledge of the molecular mechanisms associated with ageing such as chronic inflammation, DNA damage, dysfunctional mitochondria and increased senescent cell load has led to testing various therapeutic interventions [12, 13]. These interventions primarily aim to slow the ageing process and increase health span by preventing the development of chronic disease and disability [12].

There are various pharmacological interventions that have been indicated by the National Institute on Aging (NIA) Interventions Testing Program (ITP) in pre-clinical trials which target disease prevention and life span extension in rodents [14–16]. At present there is no Food and Drug Administration (FDA)-approved drug for an ageing-related indication [14]. Certain drugs such as metformin and rapamycin, which are already in clinical use, are being tested in human trials to target the ageing process to prevent development of chronic diseases [14]. The literature suggests that there are unique challenges associated with conducting such healthy ageing trials in human subjects [17–21]. Some of these challenges include lack of a consensus regarding reliable ageing biomarkers, inclusion of mainly healthy subjects in anti-ageing trials (due to multi-morbidities and medication being an exclusion criteria) and issues of participant diversity which weakens the generalisability of findings [17–21].

Moreover, the randomised controlled trials conducted have utilised a diverse range of outcome measures to determine the effectiveness of interventions, suggesting a lack of a standardised approach to measure outcomes pertaining to healthy ageing [22–24]. This makes evidence synthesis and comparisons of the effectiveness of various interventions difficult [22–24].

These issues could be addressed through the development and utilisation of a core outcome set—‘an agreed standardised set of outcomes that should be measured and reported, as a minimum, in all clinical trials in a specific area’ [25]. This can be achieved through multiple stakeholder (industry, clinicians, patients and investors) input. It is important for these key stakeholders to discuss and agree on ‘what to measure’ and ‘how to measure’ in the context of healthy ageing trials.

UK SPINE is a national knowledge exchange network composed of higher educational institutions, industry and the charitable sector which aims to promote research in healthy ageing [26]. This study is part of the UK SPINE initiative. The objectives of the study were to (i) review the outcome measures utilised in healthy ageing trials specifically focusing on medicinal drugs, dietary supplements and devices; (ii) to summarise the views of key stakeholders (industry, clinicians, patients and investors) on the outcomes that they considered most important when conducting healthy ageing trials and how these can be measured as a first step to inform future core outcome set development.

## Methods

This mixed methods study comprised of an analysis of the records of healthy ageing trials registered on the Clinicaltrials.gov database and qualitative data collected during a workshop with key stakeholders who attended the UK SPINE annual conference 2022 [26].

### Review of healthy ageing trials

#### Search strategy and selection criteria

The ClinicalTrials.gov database was searched for records of relevant studies. The database is designed to provide easy access to summary information on publicly and privately funded clinical trials and observational studies [27]. The terms ‘healthy ageing’, ‘ageing well’, ‘ageing’, ‘anti-aging’ and ‘aging process’ were searched from inception to May 2022. Studies were eligible if identified as a healthy ageing trial of pharmacological therapies, dietary supplements or medical devices. Studies were excluded if they included interventions other than pharmacological therapies, supplements and medical devices.

#### Data screening

The retrieved records were downloaded in an Extensible Markup Language (XML) file. Two independent authors (MS and OLA) systematically screened for eligible studies. Discrepancies were resolved through discussion and a third author (MC) was involved when necessary.

#### Data extraction and analysis

Data extraction occurred after the final selection of included studies. MS and OLA independently extracted details on clinical condition, type of intervention (drugs or devices), outcomes assessed, main outcome measures, interventional model, age and gender of participants, inclusion criteria of study, study funding and study location.

### Stakeholder workshop

#### Aim of the workshop

The UK SPINE annual conference 2022 was designed to explore the scientific research behind healthy ageing, including showcasing some of the research UK SPINE has supported, as well as providing a platform for discussion of the broader context for translational research to treat age-related conditions [26]. The conference therefore provided the ideal forum to engage with key stakeholders that possess the knowledge and experiences relevant to the study. A workshop was organised to explore and capture diverse perspectives from participants on several topics including the role of stakeholder groups in conducting healthy ageing trials and outcomes selection; outcomes considered most important in healthy ageing trials; challenges associated with measuring outcomes; and strategies for consensus and development of a core outcome set (COS) for healthy ageing trials testing drugs, supplements and devices interventions.

#### Recruitment of participants for the workshop and format of the workshop

Individuals registered for the UKSPINE annual conference were invited via email to participate in the workshop. The participants were provided information sheets and given the opportunity to ask questions. Written consent was taken prior to starting the workshop by researchers (MS and EM) who facilitated the workshop.

A summary of the findings of the Clinicaltrials.gov analysis was presented to the workshop participants. This was followed by interactive discussions using a topic guide which had domains pertaining to the challenges, barriers, the need to develop a COS, strategies for development of core outcome set and the role of the main stakeholders for measuring outcomes whilst testing drugs and devices interventions in healthy ageing trials (Table 1).

**Table 1:** Workshop discussion guide

• Please kindly share your professional background and experience of working or being affiliated with healthy ageing trials?
• What type of studies (interventions tested in healthy ageing trials) have you been involved with?
• In your opinion, which outcomes should be considered most important whilst testing effectiveness of pharmacological or device interventions in healthy ageing trials?
• In your opinion what are the challenges associated with measuring outcomes in healthy ageing trials?
• In your opinion, is there a need for core outcome set development for healthy ageing trials? That is an agreed standardised set of outcomes that should be measured and reported, as a minimum, in all healthy ageing trials
• If yes, please share your thoughts and opinion of why you think there is a need for core outcome set development for healthy ageing trials
• In your opinion which stakeholders should be involved in developing core outcome set development for healthy ageing trials?
• Briefly describe the strategies that can be utilised for consensus and development of a core outcome set for healthy ageing trials?

#### Data collection and analysis

Flipcharts were utilised to make note of responses of participants and highlight key points as the various themes were discussed amongst the key stakeholders. Personal reflection notes were also taken during the discussions to aid reflection and inform the analysis of the qualitative data. The workshop ran for 45 min as a breakout session within the conference.

The data was analysed using thematic analysis [28]. The data was analysed by MS and EM who interpreted explored and reported patterns and clusters of meaning within the given data [29]. This technique was selected as it allows flexibility as it is not tied to any particular discipline or set of theoretical constructs [29]. A Computer Assisted Qualitative Data Analysis (CAQDAS) package (NVivo 12 for Windows) was utilised for this process. The results of the study were reported in accordance with ‘Consolidated criteria for reporting qualitative research’ (COREQ) [30] and ‘Standards for reporting qualitative research’ (SRQR) [31] guidelines to ensure good qualitative research practice.

## Results

### Analysis of healthy ageing trials

The search on the ClinicalTrials.gov database yielded 342 studies. Of these, 36 primarily focused on testing drugs, supplements or devices in healthy ageing trials and were included in the review.

#### Demographic details and study characteristics

A majority of the studies (*n* = 31; 86%) included were interventional studies (Table 3) and just over half (*n* = 20, 55%) were from the USA (Table 2). A wide range of interventional models were utilised ranging from parallel assignment, cross over assignment to observational (Table 3). The age of the participants ranged from 13 to 100 years in the included studies (Table 3) and most included male and female participants (Table 3). Funding of 23 was generated by academia, followed by pharmaceutical industry (*n* = 5), medical manufacturing (*n* = 5), healthcare institution (*n* = 1) and multi-lateral network (pharmaceuticals, academia and non-profit organisations) (*n* = 2) as demonstrated in Table 2.

**Table 2:** Interventions and outcome measures in healthy ageing trials

Study no./study status	Study title	Main aim of the study	Condition or disease	Intervention/treatment	Main outcome/outcome measures	Sponsor/country of origin of study
1. Completed (last updated August 9, 2018) First posted: Dec 10, 2012	DO-HEALTH/Vitamin D3—Omega3—Home Exercise-Healthy ageing and Longevity Trial (DO-HEALTH)	To establish whether vitamin D, omega-3 fatty acids, and a simple home exercise program will prevent disease at older age	Improve healthy ageing in seniors; prevent disease at older age	Drug: vitamin D3 Drug: omega 3 fatty acids Procedure: strength home exercise Procedure: flexibility home exercise	i) Diagnostic (X-rays) ii) Physiological (incidence of non-vertebral fractures, lower extremity function, systolic and diastolic blood pressure) iii) Cognitive (Montreal Cognitive Assessment)	Academia, pharmaceutical and NGOs (multi-centre sponsorship, Austria, Germany, France etc.) Country of origin: Switzerland
2. Enrolling by invitation (last updated Jan 13, 2022) First posted: Aug 10, 2020	Amyloid Imaging With 11C-PiB in Healthy Ageing and Mild Cognitive Impairment	To measure the amount of amyloid in the brain. Amyloid is a protein found in the brain of patients with Alzheimer’s disease and can be detected using a positron emission tomography (PET) scan	Alzheimer disease/healthy ageing	Drug: 11C-PiB Pittsburgh compound-B (11C-PiB) is a diagnostic imaging agent that binds to beta-amyloid plaques and allows them to be viewed using positron emission tomography (PET) imaging Other name: Pittsburgh compound B	i) Diagnostic: amyloid burden	Academia (University of Colorado, Denver) Country of origin: USA
3. Recruiting (last updated 20 April 2022) First posted: May 7, 2021	Modulating the Locus Coeruleus Function	To investigate the impact of transcutaneous vagus nerve stimulation (tVNS) on specific brain regions involved in memory and attention processes	Healthy ageing	Transcutaneous vagus nerve stimulation	I) Diagnostic (pupillometry—scan) II) Physiological (measure of pupil size variations) III) Cognitive (memory tasks)	Academia (Maastricht University Medical Center) Country of origin: Netherlands
4. Completed (last updated Oct 5, 2021) First posted: April 24, 2020	Signal Propagation and Its Relationship to Cognitive Performance in the aging Human Brain (Focus or Spread)	To investigate neural dedifferentiation and its relationship to cognitive performance in the healthy aging brain	Healthy ageing	Device: single-pulse TMS Device: sham stimulation	i) Diagnostic (age-related differences in the spatio-temporal patterns of signal propagation) ii) Cognitive tests	Academia (University Hospital Inselspital, Berne) Country of origin: Switzerland
5. Not yet recruiting (last updated 5 Jan 2022) First posted: 5 Jan 2022	Evaluating the Neurocomputational Mechanisms of Explore-Exploit Decision Making in Older Adults	To evaluate the neurocomputational mechanisms of explore-exploit decision making in older adults	Healthy ageing	Device: continuous theta burst transcranial magnetic stimulation	Neurophysiological testing	Academia (University of Arizona) Country of origin: USA
6. Completed (last updated 17 March 2022) First posted: Sept 5, 2021	The Effects of Post Aerobic Exercise Hot Water Immersion on Physiological and Perceptual Responses in Physically Inactive Middle-aged Adults	To investigate whether the use of post exercise hot water immersion can prolong and/or intensify exercise mediated physiological responses that underpin health benefits, whilst also assessing the perceptual responses, in comparison to exercise and hot water immersion alone	Healthy ageing	Device: post exercise hot water immersion Device: exercise Device: hot water immersion	I) Biochemical (change in circulating plasma) II) Physiological (change in brachial artery total shear rate, systolic and diastolic blood pressure)	Academia (Coventry University) Country of origin: UK
7. Recruiting (last updated November 26, 2021) First posted: August 16, 2021	A Mobile Tai Chi Platform for Fall Prevention in Older Adults—Phase II	To test the delivery of a novel Tele-Tai Chi (TC) intervention in a single-arm feasibility study for community-dwelling TC-naïve older adults	Healthy ageing	Device: Tele-Tai Chi	III) Physiological (Tai Chi home practice sessions completed by participants)	Healthcare sector (Spaulding Rehabilitation Hospital) Country of origin: USA
8. Recruiting (last updated July 1, 2021)	Modulating Prospective Memory in Older Adults With Non-invasive Brain Stimulation	To modulate neural activity in the left and right inferior frontal lobe as well as in the right superior parietal lobe via high-definition transcranial direct current stimulation (HD-tDCS) in older adults	Healthy ageing	Device: non-invasive brain stimulation Device: sham stimulation	i) Cognitive (memory testing)	Academic (University of Bern) Country of origin: Switzerland
9. Completed (last updated March 25, 2022)	The Nicotinic Cholinergic System and Cognitive aging	To examine the role of the nicotinic system in the healthy aging brain and examine its role in memory and thinking processes in older and younger adults	Healthy ageing	Drug: nicotine patch, oral mecamylamine, placebo	i) Cognitive (age effects of nicotinic blockade)	Academic (University of Vermont) Country of origin: USA
10. Recruiting (last updated April 7, 2022)	Neuroimaging in Healthy Ageing and Senile Dementia (HASD IND) (HASD PIB IND)	To evaluate the structure and function of the brain in healthy aging and early Alzheimer’s disease using positron emission tomography (PET), magnetic resonance imaging (MRI), and computed tomography (CT) imaging	Alzheimer disease	Drug: [11C]-Pittsburgh compound B ([11C]PiB) Drug: F 18 AV-1451 (flortaucipir)	i) Diagnostic biomarkers (AD biomarkers seen on amyloid PET and MRI)	Medical professional (Tammie L. S. Benzinger, MD, PhD) Country of origin: USA
11. Recruiting (last updated March 26, 2021)	The Impact of Phosphate Metabolism on Healthy Ageing	Determine the association between duration and dose of chronic conventional therapy with Pi and renal (nephrocalcinosis/nephrolithiasis), vascular (endothelial function), and cardiovascular function (echo-cardiography) in patients with hereditary hypophosphatemic rickets with hypercalciuria (HHRH) and patients with X-linked hypophosphatemia (XLH)	Hypophosphatemia Rickets Hypercalciuria XLH HHRH	Drug: phosphate	I) Biochemical (parathyroid hormone and fibroblast growth factor 23 (FGF23) levels)	Academic (Yale University) Country of origin: USA
12. Completed (last updated 30 June 2020)	Enhancing Physical Activity and Healthy Ageing Among Recent Retirees (REACT)	To provide cost-effective way to promote physical activity and reduce sedentary time among older adults	Exercise Ageing Retirement	Device: Polar Loop 2 activity tracker	I) Physiological (change in wake time physical activity, systolic and diastolic blood pressure; body composition: fat mass, fat free mass, body weight, waist circumference) II) Biochemical (fasting blood sample: lipid profile (total cholesterol, HDL, LDL, triglycerides), fasting plasma glucose, HbA1C, and CRP)	Academic (University of Turku) Country of origin: Finland
13. Unknown (last updated May 21, 2018)	Effects of High-velocity Resistance Training and Creatine Supplementation in Healthy Ageing Males	To compare the effects of HVRT and CR supplementation to HVRT and placebo in healthy aging males	Sarcopenia	Drug: creatine monohydrate Drug: maltodextrin powder	i) Physiological (change in muscle thickness change in muscle strength, walking speed)	Academic (University of Regina) Country of origin: Canada
14. Suspended	Effects of Methylene Blue in Healthy Ageing Mild Cognitive Impairment and Alzheimer’s Disease (MB2)	To investigate the effect of 2-week and 12-week administration of USP methylene blue (MB) on cerebral blood flow, functional connectivity, memory and attention cognitive abilities using fMRI and behavioural measures in healthy aging, mild cognitive impairment (MCI) and mild Alzheimer’s disease (AD) subjects	Mild cognitive impairment Aging Alzheimer’s disease	Drug: methylene blue Drug: FD&C Blue # 2 Drug: phenazopyridine hydrochloride	I) Cognitive (working memory task) II) Diagnostic (MRI) III) Physiological (cerebral blood flow measures)	Academic (The University of Texas Health Science Center at San Antonio) Country of origin: USA
15. Recruiting (last updated Sept 2, 2021)	Novel, Individualized Brain Stimulation, Network-based Approaches to Improve Cognition (NiBS-iCog)	To investigate dynamic interactions between distinct cortico-cortical and subcortico-cortical brain areas	Mild cognitive impairment Healthy ageing	Device: tACS Device: TMS	i) Cognitive (working memory performance)	Academic (Masaryk University) Country of origin: Czech Republic
16. Recruiting (last updated August 30, 2021)	Cause-effect Relationships Between Brain Networks and Bimanual Coordination in Older Adults	To investigate whether high-definition dual-site transcranial alternating current stimulation can improve bimanual motor learning in healthy older adults	Healthy aging	Device: HD-tACS Device: sham HD-tACS	i) Cognitive (bimanual coordination accuracy during the stimulation or sham and working memory during stimulation or sham)	Academic (Hasselt University) Country of origin: Belgium
17. Recruiting last updated May 2, 2022	Telerehabilitation Alzheimer’s Disease Feasibility	To investigate whether therapeutic games improve the memory cognitive domain	Alzheimer disease Healthy aging	Device: BrightGo cognitive training Drug: standard of care medication for early Alzheimer’s disease	i) Neuropsychological Assessment Battery ii) Cognitive (memory tests) iii) Mental health (depression states)	Medical equipment manufacturing company (Bright Cloud International Corp) Country of origin: USA
18. Recruiting (last updated August 27, 2021)	A Novel Computer-Based Functional Skills Assessment and Training Program	To evaluate a novel integrated computer-based functional skills assessment and training (CFSAT) program that provides training on everyday tasks critical to independent living (e.g., financial and medication management) with non-impaired older adults (NC) and adults with MCI	Mild cognitive impairment Healthy aging	Device: The Computerized Functional Skills Assessment and Training Program Device: Brain HQ Double Decision	I) Cognitive (time to completion on each tasks)	Medical devices company (i-Function, Inc) Country of origin: USA
19. Not yet recruiting (last updated March 14, 2022)	Electromagnetic Field Protection Device Use Impact in Healthy Volunteers	To evaluate the clinical and molecular impact of continuous in-home resonance-based electromagnetic field (EMF) protection device usage in healthy individuals	Aging well	Device: in-home resonance-based electromagnetic field protection device	i) Diagnostic: experimental: in-home EMF protection device	TruDiagnostic (medical device company) Country of origin: USA
20. Completed (last updated Feb 24, 2021)	The Effect of Transcranial Direct Current Stimulation on Visual Attention—Single Sessions	To examine the short-term effects of multiple tDCS protocols in healthy adult population on visual attention and identify the neural underpinnings of tDCS-induced behavioral aftereffects using a combined tDCS/ MRI network-based approach	Healthy aging Cognitive change Cognitive decline	Device: transcranial direct current stimulation	i) Cognitive (visual-attention task accuracy)	Academic (Masaryk University) Country of origin: Czech Republic
21. Not yet recruiting (last updated April 27, 2022)	Development of Physical Activity Features for Ear-worn Devices	Development of software features related to physical activity for users of ear-worn devices	Hearing loss Well aging	Device: ear-worn prototype devices	i) Diagnostic (accuracy of ear-worn device)	Medical devices company (57GGardner) Country of origin: USA
22. Unknown (last updated, 26 March 2018)	Neural Correlates of Repeated tDCS	To investigate the neural effects of repeated transcranial direct current stimulation in ameliorating mild cognitive impairment	Mild cognitive impairment	Device: active tDCS Device: sham tDCS	I) Cognitive (neural changes after multi-session tDCS)	Academic (Maastricht University Medical Center) Country of origin: Netherlands
23. Completed (last updated April 22, 2022)	Evaluation of the Benefits for Overall Health Following Cochlear Implant Treatment in the Elderly Population	To show that cochlear implant treatment improves the overall health-related quality of life and general well-being in elderly individuals	Hearing loss	Device: Commercial Nucleus Cochlear Implant Systems	i) Patient reported outcome (change in health-related quality of life following cochlear implant treatment)	Medical device company (Cochlear) Country of origin: France, Israel etc. (multi-country)
24. Recruiting (last updated May 2, 2022)	SHAPES: Supporting Multimorbid Older People	To identify, manage and improve deficiencies in adherence to medicines and treatments of older individuals living with permanent or temporary reduced functions or capabilities due to chronic, age-related illnesses and living at home	Heart failure Diabetes Diabetes mellitus	Device: SHAPES app	i) Cognitive (participants’ engagement with the SHAPES app during the pilot)	Academic (Prof Michael Scott) Country of origin: UK
25. Completed (last updated)	Xanamem™ in Healthy Elderly Subjects (XanaHES)	To test the safety and tolerability of Xanamem	Safety Peripheral neuropathy Cortisol Central nervous system Cognitive function Healthy ageing Small fibre neuropathy Cerebrospinal fluid Electrocardiography	Drug: Xanamem Drug: matching placebo	I) Diagnostic (incidence of treatment-emergent adverse events (AEs), ECG etc., clinically significant changes in serum biomarker levels in a standard serum chemistry panel, nerve conduction assessments will be used to detect presence and severity of nerve damage, skin biopsy etc.) ii) Cognitive (quantitative sensory testing (QST))	Pharmaceutical industry (Actinogen Medical) Country of origin: Australia
26. Not yet recruiting (last updated Sept 21, 2021)	Electronic-Nutrition-Optimizer for Personalized Prevention (eNO)	To develop and validate a tool for immediate nutrition assessment and to test its user feasibility in routine clinical practice for health promotion	Diabetes Hypertension Frailty Dementia Cardiovascular diseases	Device: eNutrition Optimizer	i) Cognitive (perception questionnaire using system usability scale)	Academic (Heike Bischoff-Ferrari) Country of origin: Switzerland
27. Recruiting (last updated Oct 5, 2021)	Participatory Evaluation (of) Ageing (With) Rapamycin (for) Longevity Study (PEARL)	To establish a long-term safety profile, determine the long-term efficacy of rapamycin in reducing clinical aging measures, and biochemical and physiological endpoints associated with declining health and aging in healthy older adults	Aging	Drug: rapamycin Drug: placebo	i) Diagnostic (changes in visceral fat as measured by dual-energy X-ray absorptiometry (DXA) scan)	Pharmaceutical (AgelessRX) Country of origin: USA
28. Completed (last updated April 5, 2017)	This is a Study to Evaluate the Effect of Aging of Multiple Doses of GLPG1205 in Healthy Subjects	To evaluate the effect of aging on safety, tolerability and PK of multiple oral doses of GLPG1205 in healthy male subjects	Healthy Elderly	Drug: GLPG1205 50 mg q.d Drug: placebo oral capsule Drug: GLPG1205 250 loading dose and 50 mg q.d. maintenance dose	I) Physiological (physical examination) II) Biochemical (laboratory investigations)	Pharmaceutical (Galapagos NV) Country of origin: Belgium
29. Completed (last updated April 1, 2019)	Phase 1 Study of the Effects of Combining Topical FDA-approved Drugs on Age-related Pathways on the Skin of Healthy Volunteers	To examine the effects of 3 FDA-approved medications on skin aging when applied in topical form	Aging	Drug: sirolimus Drug: metformin Drug: diclofenac	i) Diagnostic (profile of gene transcript changes and wrinkle score +)	Academic (Anne Chang) Country of origin: USA
30. (Last updated April 13, 2017)	The Effects of Aging and Estrogen on the Pituitary	To study the effects of aging and estrogen on the brain	Healthy	Drug: GnRH Drug: NAL-GLU GnRH antagonist Drug: estrogen patch	I) Biochemical (pituitary response to GnRH and effect of estrogen on pituitary response to GnRH)	Academic (Massachusetts General Hospital) Country of origin: USA
31. Completed (last updated Dec 9, 2019)	Brain Blood Flow: Age and Gender	To employ advanced neuroimaging techniques to enhance the investigators understanding of neurovascular coupling in healthy aging	Healthy Aging	Device: MRI Device: TCD	I) Physiological (middle cerebral artery flow measurement)	Academic (University of Wisconsin, Madison) Country of origin: USA
32. Recruiting (last updated March 19, 2021)	Non-invasive Nerve Stimulation and Cognitive Training to Improve Cognitive Performance in Healthy Older Adults	To evaluate the pairing of cognitive training with a non-invasive neurostimulation technology that has shown promise in both increasing neuroplasticity and in enhancing cognitive performance, transcutaneous vagal nerve stimulation (tVNS)	Aging	Device: transcutaneous vagal nerve stimulation (tVNS)	I) Cognitive (change in working memory and processing speed contributors)	Academic (University of Florida) Country of origin: USA
33. Completed (last updated November, 20, 2017)	Pilot Investigation of a Multinutrient Supplement on Skin Aging and Aging Metabolites in Healthy Women	To determine if multinutrient supplementation affects visible signs of skin aging as well as blood measurements of aging. We are seeking smokers and non-smokers	Skin diseases	Drug: dietary supplement: LifePak Nano	I) Biochemical (fold change in long chain fatty acids) II) Diagnostic (change in fine wrinkling)	Academic (Stanford University) Country of origin: USA
34. Completed (last updated March 23, 2018)	A Clinical Study to Investigate the Effects of Creatine Supplementation on Muscle Energetics and Cognitive Function in Young Healthy Male Athletes and an Ageing Population	To examine the impact of creatine supplementation on muscle function and cognitive performance in young and older subjects	Nutritional status	Drug: creatine Drug: placebo	I) Diagnostic (PCr concentration (at rest))	Pharmaceutical (GlaxoSmithKline) Country of origin: UK
35. Active, not recruiting (last updated August 3, 2021)	Safety and Effectiveness of Quercetin & Dasatinib on Epigenetic Ageing	To assess the effects of quercetin and dasatinib over a 16-week period on participants’ epigenetic biological ageing	Aging	Drug: dasatinib plus quercetin	i) Physiological (epigenetic age test) ii) Diagnostic (examining methylation changes [time frame: change from baseline to 6 months])	Pharmaceutical industry (TruDiagnostic) Country of origin: USA
36. Active (recruiting)	Targeting aging with Metformin (TAME)	To assess the ability of metformin to slow aging in humans	Aging	Metformin	Progression to an age-related disease (cardiovascular disease, cancer, cognitive disease) or mortality in an at risk group of non-diabetics	NGO (American Federation for Ageing Research) Country of origin: USA

**Table 3:** Study and patient characteristics in healthy ageing trials

Study no	Study type/study phase	No. of participants	Interventional model	Sex/gender of participants	Age of participants	Inclusion criteria (main indicators considered)
1	Interventional (clinical trial) phase 3	2157 participants	Factorial assignment	Sexes eligible for study: all	70 years and older	Age, mental health, mobility, cognitive and physiological function
2	Observational (case control)	200 participants	Observational model	Sexes eligible for study: all	50 years and older	Age, cognitive function and previous participation in trial and ability to tolerate long duration of PT scan
3	Observational	30 participants	Observational model	Sexes eligible for study: all	60 to 80 years	Average neurophysiological tests, BMI, smoking status and cognitive function
4	Interventional (clinical trial)	40 participants	Parallel assignment	Sexes eligible for study: all	20 to 75 years (adult, older adult)	Age, neurologically healthy and normal or corrected-to-normal visual acuity
5	Interventional (clinical trial)	240 participants	Cross over assignment	Sexes eligible for study: all	18 to 80 years (adult, older adults)	Age, cognitive and physiological function
6	Interventional (clinical trial)	16 participants	Cross over assignment	Sexes eligible for study: all	40 to 60 years (adults)	Age, BMI, maximal oxygen uptake
7	Interventional (clinical trial)/phase 2	30 participants	Single group assignment		60 to 85 years (older adults)	Age, computer literacy, physiological function (ability to walk continuously for 15 min without an assistance)
8	Interventional (clinical trial)	105 participants	Parallel assignment	Sexes eligible for study: all	60 to 80 years (older adults)	Cognitive function (no cognitive impairments), German speaking, right-handed, normal, or corrected-to-normal vision, non-smokers
9	Interventional (clinical trial)	96 participants	Parallel assignment	Sexes eligible for study: all	18 to 75 years (adult, older adults)	Cognitive functions (normal cognition, not demented, no mild cognitive impairment. IQ greater than 80)
10	Observational	650 participants	Observational model (other)	Sexes eligible for study: all	18 years and older (adult, older adults)	Age, cognitive function and previous history of participation
11	Interventional (clinical trial)	30 participants	Parallel assignment	Sexes eligible for study: all	13 years and older (child, adult, older adults)	Age and genetic predisposition
12	Interventional (clinical trial)	231 participants	Parallel assignment	Sexes eligible for study: all	60 to 65 years (adult, older adults)	Physiological functionality and computer literacy
13	Interventional (clinical trial)	40 participants	Parallel assignment	Male	50 years and older (adult, older adults)	Age and physiological functionality
14	Interventional (clinical trial)	117 participants	Single group assignment	Sexes eligible for study: all	45 to 89 years (adult, older adults)	Age, physiological function and condition specific (Alzheimer’s early-stage, sporadic-type)
15	Interventional (clinical trial)	160 participants	Crossover assignment	Sexes eligible for study: all	60 years and older (older adults)	Cognitive function
16	Interventional (clinical trial)	100 participants	Crossover assignment	Sexes eligible for study: all	65 to 77 years (older adults)	Cognitive (> 25 on Montreal Cognitive Assessment Questionnaire) and physiological function
17	Interventional (clinical trial)	14 participants	Crossover assignment	Sexes eligible for study: all	65 to 85 years (older adults)	Age, cognitive (e.g. Montreal Cognitive Assessment and physiological function (good upper extremity motor function, close to full range of movement of arms and fingers))
18	Interventional (clinical trial)	180 participants	Parallel assignment	Sexes eligible for study: all	65 to 100 years (older adults)	Cognitive function and intervention specific eligibility
19	Interventional (clinical trial)	44 participants	Single group assignment	Sexes eligible for study: all	30 to 70 years (adults, older adults)	Age and intervention specific eligibility
20	Interventional (clinical trial)	50 participants	Crossover assignment	Sexes eligible for study: all	18 to 80 years (adults, older adults)	Age and physiological function (e.g. mobility)
21	Interventional (clinical trial)	20 participants	Single group assignment	Sexes eligible for study: all	40 to 85 years (adults, older adults)	Age and intervention-related eligibility
22	Interventional (clinical trial)	80 participants	Parallel assignment	Sexes eligible for study: all	60 to 85 years (older adults)	Age, physiological function and intervention-based eligibility
23	Observational	100 participants	Observational model (cohort)	Sexes eligible for study: all	60 years and older (older adults)	Age, cognitive function and intervention-based eligibility
24	Interventional (clinical trial)	30 participants	Single group assignment	Sexes eligible for study: all	60 to 100 years (older adults)	Age and intervention-based eligibility
25	Interventional (clinical trial)	42 participants	Parallel assignment	Sexes eligible for study: all	50 to 75 years (adults, older adults)	Age, physiological function and intervention-based eligibility
26	Interventional (clinical trial)	150 participants	Crossover assignment	Sexes eligible for study: all	50 years and older (adults, older adults)	Age, intervention-based eligibility
27	Interventional (clinical trial)	150 participants	Parallel assignment	Sexes eligible for study: all	50 to 85 years (adults, older adults)	Age, intervention-based eligibility
28	Interventional (clinical trial)	32 Participants	Parallel assignment	Male	18 years and older (adults, older adults)	Age, physiological function and intervention-based eligibility
29	Interventional (clinical trial)	10 participants	Sequential assignment	Female	55 years and older (adults, older adults)	Age, physiological function and intervention-based eligibility
30	Interventional (clinical trial)	19 participants	Sequential assignment	Female	45 to 80 years (adults, older adults)	Age, physiological function and intervention-based eligibility
31	Observational	36 participants	Observational model (case–control)	Sexes eligible for study: all	20 to 64 years (adults, older adults)	Age and physiological function
32	Interventional (clinical trial)	40 participants	Parallel assignment	Sexes eligible for study: all	65 to 90 years (adults, older adults)	Physiological function and intervention-based eligibility
33	Interventional (clinical trial)	37 participants	Parallel assignment	Female	18 to 70 years (adults, older adults)	Age, physiological function and intervention-based eligibility
34	Interventional (clinical trial)	30 participants	Parallel assignment	Sexes eligible for study: all	18 to 70 years (adults, older adults)	Age, physiological function and intervention-based eligibility
35	Interventional (clinical trial)	25 participants	Single group assignment	Sexes eligible for study: all	40 years and older (adults, older adults)	Age and intervention-based eligibility
36	Interventional (clinical trial)	3000 participants	Parallel assignment	Sexes eligible for study: all	65 to 79 years	Age and intervention-based eligibility

#### Conditions considered in the studies

**Fig. 1:**
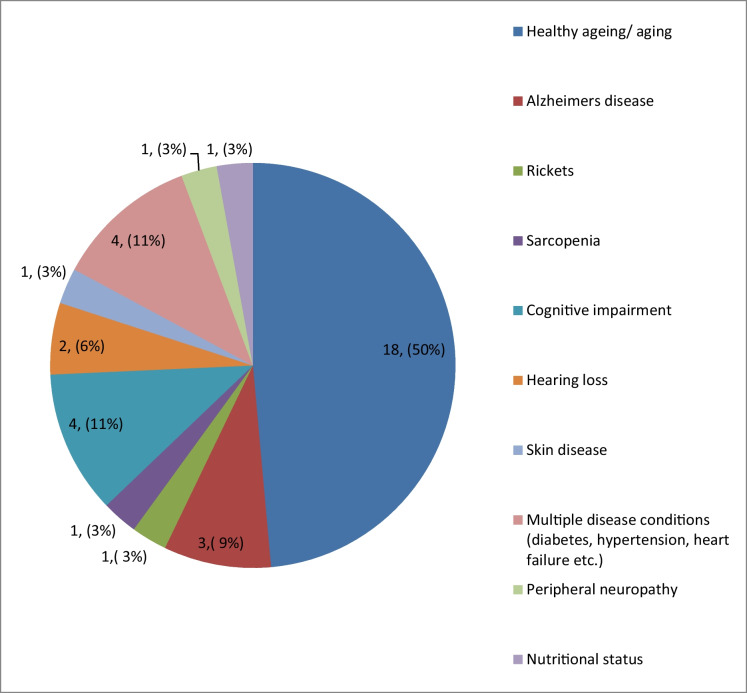
Type of disease conditions addressed in studies (*n* = 36)

The prevention of a range of diseases was the primary goal of the studies. Half of the studies (50%) investigated general ‘healthy ageing’ whilst others focused on prevention of specific disease conditions such as cognitive impairment, Alzheimer’s disease, hearing loss and multiple disease conditions (Table 2 and Fig. 1).

#### Interventions

There was marked heterogeneity in the interventions evaluated in the studies as shown in Table 2. Pharmacological agents investigated included vitamin D3, omega 3 fatty acids, phosphate, creatine monohydrate, maltodextrin powder etc. (Table 2). Devices investigated included Tele-Tai Chi, non-invasive brain stimulation and in-home resonance-based electromagnetic field protection device (Table 2).

#### Outcome measures utilised in studies

In total, 15 studies included cognitive outcome measures such as working memory task and performance (Table 2). Other outcome measures included diagnostic (biomarkers and lab tests) (*n* = 12), physiological (pupil size, muscle thickness, physical activity etc.) (*n* = 12), biochemical (blood oxygen levels, plasma nitrates, hormone levels etc.) (*n* = 5), mental health (e.g. Beck’s inventory) (*n* = 1) and patient-reported outcome measure (*n* = 1) as depicted in Table 2.

### Stakeholder workshop

#### Demographic details and themes identified from the workshop

There were a total of 10 participants that attended the workshop. The workshop participants had diverse professional backgrounds including academics (*n* = 2), investors (*n* = 2), representatives of pharmaceutical industry (*n* = 2), clinician (*n* = 1), patient and public involvement and engagement (PPIE) manager (*n* = 1), patient (*n* = 1) and policy maker (*n* = 1). Their experience of healthy ageing trials ranged from 1 to 5 years. The following themes were identified (Table 4):

i) Role of stakeholder groups
  A central finding of the workshop was the acknowledgement that whilst academia and the pharmaceutical industry commonly take the lead in healthy ageing trials, a larger number of stakeholders should be involved. A PPIE manager elaborated about the significance of academics and pharmaceutical industry professionals initiating healthy ageing research:
  *‘Academia and pharmaceuticals have the required knowledge and skills to conduct research. Not everyone can understand the biological process involved in ageing and how it can be intervened. They should lead firstly and then engage and involve other key stakeholders’*

**Table 4:** Thematic analysis of workshop discussions

Theme	Subtheme	Quotes
Role of stakeholders	Responsibility of academia and pharma industry	*‘Academia and pharmaceuticals have the required knowledge and skills to conduct research. Not everyone can understand the biological process involved in ageing and how it can be intervened. They should lead firstly and then engage and involve other key stakeholders’*
	Collaborative approach	*‘I think it should be a collaborative approach, the academia needs to generate the evidence which can be shared with the key stakeholders such as industry, policy makers and even patients and their engagement officers while designing aging trials considering the multi-morbidities in the elderly’*
Important outcomes to consider	Essential outcomes	*‘In my opinion mobility, autonomy and quality of life measures are extremely important to consider especially from the perspective of patients’*
	Other outcomes	*‘Outcomes such as diagnostic which may include lab tests and biomarkers should be carefully considered and I think…assessment of physiological indicators are also very important’*
Challenges associated with measuring outcomes	Co-morbidities and specific interventions	*‘There are challenges in teasing out the right outcomes due to the intervention that is selected and I would say that the comorbidities developed in ageing further complicates it’* *‘As elderly patients generally have multiple morbidities, polypharmacy and associated side effects of existing drug prescriptions can influence the outcomes that are assessed of a particular intervention. The findings can be biased and not truly representative of the effectiveness of the drug. It is worth considering the complexity of chronic disease and its treatment among the elderly patients’*
	Participant recruitment and retention	*‘Most trials exclude elderly patients with multiple diseases, it is important to recruit a representable sample and make the findings generalizable… another concern I would say is that such trials are lengthy and recruiting and retaining elderly population in such trials can be challenging. You can’t overlook dropouts as well, this also has to be taken into consideration’*
	Powering of trials	*‘While designing trials powering of trials to get information related to novel biomarkers can be difficult and if we don’t get it right it can influence the findings of the study and its interpretation’*
	Attributing outcomes to a set of measures	*‘It is important to mention that attributing outcomes to a set of measures that provide information about the drug mechanism and the ageing biology being modulated can be challenging… when dealing with pharmacological interventions…it can always be difficult to get this right… careful consideration is required I would say’*
Strategies for establishing core outcomes	Building consensus	*‘Probably we can aid consensus between trials testing different pathways or mechanisms of ageing biology, particularly in the current… absence of ageing being a registerable endpoint itself. This would aid development of novel therapeutics and biomarkers, giving researchers a common, testable, framework, and allow comparison between outcomes’* *‘Diverse representation from population groups in terms of gender, ethnicity and socioeconomic backgrounds should be involved and engaged in consensus building exercise’* *‘Perhaps a staged approach taking the views of each would be helpful to set out the initial thoughts…concerns prior to the design of an initial consultation document that can be iterated. It will be important to take into account similar efforts in other territories…countries and by other stakeholders’*
	Building on existing evidence	*‘The subject matter experts such as researchers and academics who have the relevant and substantial experience in healthy ageing trials should lead the discussions, but future design should be informed by wide consultation, particularly representative of the target population for healthy ageing trials, this I think will be the right approach’*
	Focus groups and Delphi approaches	*‘I think at later stages of development focus group discussions and Delphi approaches are very useful methods which can be utilized for refinement and consensus of mutually-agreed core outcomes set’*

An academic whilst emphasising the need to adopt a collaborative approach for designing health ageing trials commented:

*‘I think it should be a collaborative approach, the academia needs to generate the evidence which can be shared with the key stakeholders such as industry, policy makers and even patients and their engagement officers while designing aging trials considering the multi-morbidities in the elderly’*

ii) Important outcomes to consider in trials
  Various outcomes were identified as important whilst testing drugs and devices in healthy ageing trials. These included mobility, autonomy, quality of life measures, diagnostic (lab tests and biomarkers) and assessment of physiological indicators. It was further emphasised by the participants that it was important to develop consensus amongst the key stakeholders whilst selecting the outcomes to fully assess the interventions and its effects on the life span and health span of patients.
  Similarly, a policy maker highlighted the patients’ perspectives whilst considering outcomes:
  *‘In my opinion mobility, autonomy and quality of life measures are extremely important to consider especially from the perspective of patients’*
iii) Challenges associated with measuring outcomes
  Recruitment of participants and the confounding effects of multi-morbidity in older adults were identified as key challenge of measuring outcomes in healthy ageing trials. In particular, the recruitment of cohorts which are representative of the general population, including (but not limited to) ethnic minority groups at greater risk of age-related diseases. Statistical powering of studies was a major hurdle to address. Retention of participants and providing ongoing support were also critical factors for consideration as suggested by participants.

A pharmaceutical industry representative elaborated on the challenges associated with measuring outcomes in healthy ageing trials:

*‘As elderly patients generally have multiple morbidities, polypharmacy and associated side effects of existing drug prescriptions can influence the outcomes that are assessed of a particular intervention. The findings can be biased and not truly representative of the effectiveness of the drug. It is worth considering the complexity of chronic disease and its treatment among the elderly patients’*

A senior academic mentioned the challenge of patient recruitment and retention:

*‘Most trials exclude elderly patients with multiple diseases, it is important to recruit a representable sample and make the findings generalizable… another concern I would say is that such trials are lengthy and recruiting and retaining elderly population in such trials can be challenging. You can’t overlook dropouts as well; this also has to be taken into consideration’*

iv) Suggested strategies for establishing core outcomes
  Different strategies were suggested for establishing a core outcome set for healthy ageing trials. It was suggested that the evidence generated from previous published literature can serve as an important starting point to build consensus amongst the key stakeholders. It was further emphasised that academics should take a lead in such discussions. An academic commented:
  *‘The subject matter experts such as researchers and academics who have the relevant and substantial experience in healthy ageing trials should lead the discussions, but future design should be informed by wide consultation, particularly representative of the target population for healthy ageing trials, this I think will be the right approach’*

The significance of a diverse population in consensus building exercise was highlighted by the PPIE manager who suggested:

*‘Diverse representation from population groups in terms of gender, ethnicity and socioeconomic backgrounds should be involved and engaged in consensus building exercise’*

A pharmaceutical representative whilst discussing further refinement and development of mutually agreed COS suggested:

*‘I think at later stages of development focus group discussions and Delphi approaches are very useful methods which can be utilized for refinement and consensus of mutually-agreed core outcomes set’*

## Discussion

### Key findings

The main findings of the review highlight that there are a range of measures that are being utilised in healthy ageing trials testing pharmacological therapies, supplements and medical devices. However, even when addressing a similar concept there is no standardised approach to assessment of these outcomes. A range of outcome measures were considered important by workshop participants for healthy ageing trials which included mobility, autonomy, quality of life measures, diagnostic (lab tests and biomarkers) and assessment of physiological indicators. It was discussed that recruitment of participants and the confounding effects of multi-morbidity in older adults act as key challenges of measuring outcomes in healthy ageing trials. The analysis of data from the workshop highlighted the potential value of reaching consensus on a COS that could be assessed in future healthy ageing trials. Different strategies were suggested for development of COS which included gathering information from previous published literature, active involvement of key stakeholders in discussing key findings and conducting focus group discussion and use of Delphi methodology for further refinement and mutual agreement on consensus of COS.

### Comparison of study findings with existing evidence

Presently there is a dearth of biomarker and resource development to investigate novel biomarkers and therapeutic interventions [32]. There is also a need to validate the existing biomarkers that are currently being utilised including composites of different markers for utilisation in clinical trials related to ageing [13, 32]. Moreover, the literature suggests that outcomes associated with healthy ageing should be multidimensional positive health outcomes and comprehensively measure the functionality of individuals and adaption to environmental challenges in context with their social, mental and physical well-being [33]. Similarly, the World Health Organisation also strongly advocates health as a holistic concept inclusive of the essential domains of social, physical and mental wellbeing [33]. The studies included in the review utilised some of the outcomes measures based on these broader domains to assess the effectiveness of interventions (drugs or devices) in healthy ageing and other age-related diseases but there was marked heterogeneity in measures used in these trials. The diverse range of outcomes, criteria and scales for measurement of effectiveness of interventions utilised in the studies suggest a lack of a standardised approach to measure outcomes pertaining to healthy ageing. This makes comparisons of the effectiveness of interventions difficult. It can be further argued that even within individual trials focusing on limited outcome measures may not fully capture the benefits of the drug or device intervention with regard to its contribution both towards the health span and life span of patients [34–36]. Importantly, it is increasingly recognised over the last few decades that life span and health span are different attributes associated with health outcomes of interventions and cannot be interchangeably used to interpret study findings [35].

The participants recruited in the studies included in the review included healthy individuals with a certain level of physiological functionality and neurocognitive abilities. It can be argued that inclusion of only healthy older adults and excluding individuals with co-morbidities and multiple drug prescriptions can influence the effects of interventions targeting ageing making them less relevant to the older population [13].

The findings of the review and workshop highlight the significance of development of a core outcome set for healthy ageing trials. This is substantiated by the fact that there is increased emphasis in recent literature on utilisation of common data elements and core outcome measures to address the issues of interpretation, generalisation and implementation of the research findings of clinical trials [37]. Moreover, the funding bodies and regulatory agencies also strongly advocate the standardisation of reporting and conducting clinical trials [38]. The workshop participants (academics, clinicians, patient public representative, industry representatives and patient) recommended the need for multiple stakeholders to collaborate and design healthy ageing trials and develop core outcome set with special consideration of complications of co-morbidities amongst older patients. This finding is supported by evidence which describes the key challenges associated with measuring healthy ageing suggesting a multi-sectoral approach whilst designing such trials [39, 40]. The findings of this study suggest that one of the main challenges associated with measuring outcomes in healthy ageing trials is the recruitment of participants who are truly representative of the general population. Various studies conducted in developed countries support these findings and highlight the complexities of diverse socio-demographic characteristics, multi-morbidities and the prevailing health inequalities amongst the ageing population [39, 41, 42]. Hence, it is equally important to include individuals from diverse backgrounds in COS development as emphasised in the workshop by the key stakeholders.

### Limitations of the study

The limitations of the review include that there are certain limitations associated with the search engine clinicaltrials.org such as the search engine not having all information on studies and records can be changed by a responsible party. Nevertheless, it is very unlikely that the researchers would change the intervention (drugs or devices) and the set outcome measures once the study has been initiated. The review only focused on pharmacological therapies, supplements and medical devices and did not include other interventions such as behavioural, dietary or social interventions. Another limitation of the study can be that in-depth qualitative interviews were not conducted to capture views of key stakeholders. This methodology can yield more information about the perceptions of the key stakeholders and offer a more robust analysis opportunity. However, the workshop was designed to answer focused themes/questions and provided efficient opportunity to extract important information from a wide range of key stakeholders which included academics, clinicians, patient public involvement manager, industry representative and patient. The workshop was conducted as part of conference breakout, which limited participation to conference attendees. Findings from the workshop were very important, and including a diverse range of relevant stakeholders however it would have been useful to include a greater number of participants to corroborate these views.

### Future recommendations

The following key recommendations are suggested based on the findings of the study:

**Table:** 

• There is a need for core outcome set development for healthy ageing trials (for comparability across interventions and within different settings to better interpret and increase the generalisability of the findings of the studies) by active collaboration and evidence-based consensus with key stakeholders in accordance with the COMET initiative [25]
• The multi-morbidities associated chronic health complications, ongoing treatments associated with ageing should be carefully considered whilst testing pharmacological therapies, supplements and medical device interventions
• Future studies should aim to recruit participants from diverse backgrounds to increase the generalisability of findings

## Conclusion

A range of outcome measures have been used to support endpoint assessment in healthy ageing trials. The diverse range of outcomes, criteria and scales for measurement of effectiveness of interventions utilised in these studies suggest a lack of a standardised approach to measure outcomes pertaining to healthy ageing. This makes comparisons of the effectiveness of interventions difficult. The workshop provided an important platform to garner a range of perspectives on the considerations around the use of outcome measures in clinical trials for healthy ageing. It is critical that such discussions occur to progress this field and provide practical answers to how trials of this type are designed and structured.
